# A zebrafish gene with sequence similarities to human uromodulin and GP2 displays extensive evolutionary diversification among teleost and confers resistance to bacterial infection

**DOI:** 10.1016/j.heliyon.2024.e37510

**Published:** 2024-09-06

**Authors:** Shiori Naruoka, Souhei Sakata, Shigeru Kawabata, Yasuyuki Hashiguchi, Eriko Daikoku, Shoichi Sakaguchi, Fumiyoshi Okazaki, Kento Yoshikawa, John F. Rawls, Takashi Nakano, Yoshinobu Hirose, Fumihito Ono

**Affiliations:** a Department of Physiology, Faculty of Medicine, Osaka Medical and Pharmaceutical University, Japan; b Department of Pathology, Faculty of Medicine, Osaka Medical and Pharmaceutical University, Japan; c Department of Biology, Faculty of Medicine, Osaka Medical and Pharmaceutical University, Japan; d Department of Microbiology, Faculty of Medicine, Osaka Medical and Pharmaceutical University, Japan; e Department of Life Sciences, Graduate School of Bioresources, Mie University, Japan; f Department of Molecular Genetics & Microbiology, Duke Microbiome Center, Duke University School of Medicine, USA

## Abstract

In the process of investigating synaptic changes happening to mutants lacking postsynaptic receptors in the neuromuscular junction, we focused on a hitherto uncharacterized zebrafish gene *zgc153932* whose expression was increased in the RNAseq and droplet digital PCR (ddPCR) analysis of a paralyzed mutant *sofa potato*. The *zgc153932* gene which we named *omcin5 (omc5)* showed amino acid sequence similarity to human *uromodulin* and *GP2*, which are expressed in epithelial cells of the kidney and the gut respectively and bind to bacteria pili. *omc5* had 14 paralogues in a ∼400 KB region on the chromosome 12 of the zebrafish genome. These genes, named *omcin1* through *15*, constitute a gene cluster which presumably arose from recent gene duplications in the zebrafish lineage. An antibody raised against the epitope common to 6–9 genes in the *omcin* family revealed expression in the cloaca of 1 day post fertilization (dpf) embryos which broadened to the urinary and digestive tracts by 5 dpf. Expression of *omc5* was increased by exposure of embryos to *Escherichia coli (E. coli)*. Survival of *omc5* mutant embryos was shortened in the presence of *E. coli*, or when they were not maintained in germ-free conditions. Adults *omc5* mutants also exhibited susceptibility to infection. Other teleost species which had *omcin*-like genes in their genomes showed a range of gene duplication, resulting in clusters of 1 to >15 *omcin*-like genes. We hereby identified a new gene family specific to teleost that include a microbial induced gene which confers resistance to bacterial infection.

## 1 Introduction

Zebrafish is a small freshwater fish widely employed as an experimental system to study numerous biological processes, including the immunological system to combat microorganisms [[1], [2], [3], [4], [5], [6], [7], [8], [9], [10], [11], [12]]. Fish employ natural as well as acquired immunity to handle pathogens, and the molecules utilized in the defense are common between fish and mammals: e.g. immunoglobulins, MHC products, recombination activating genes, or TLRs [13]. Multiple microorganisms including *Escherichia coli* (*E. coli)* [6]*, Shigella* [3,7]*, Aeromonas* [12]*, Salmonella* [4] or *Mycobacterium* [11] can infect zebrafish, which researchers have used as an infection model [12]. In addition to elucidating the mechanism of immunity against bacterial infection conserved among vertebrates, knowledge obtained from these studies are also applicable to other areas, such as infection control in laboratories or aquaculture [14].

In the current study, we focus on a zebrafish gene, *zgc153932*, whose expression was increased in the paralyzed mutant *sofa potato (sop)* [15]. While *zgc153932* does not have a matching homolog in the mammalian genomes, it showed sequence similarity to *uromodulin* and *Glycoprotein2 (GP2)*, which are epithelial proteins controlling bacterial infection [16,17]. A group of genes including *zgc153932* underwent robust gene duplications in the recent evolutionary process and constitutes a gene cluster in the zebrafish genome. Moreover, this newly identified gene family was also conserved in other teleost species, displaying a wide variety of diversification. We explored the possibility that these genes play roles in the response of fishes to the microorganisms.

## 2 Results

### 2.1 Expression of uncharacterized gene *zgc153932* upregulated in paralyzed zebrafish mutant

*Sop* is a mutant zebrafish line, whose synaptic transmission at the neuromuscular junction is absent due to a point mutation in the δ subunit gene of the acetylcholine receptor (AChR), leading to paralysis of its skeletal muscles [18]. To explore genetic changes in synapses caused by the lack of synaptic transmission, we performed RNA-seq analysis of normal siblings (*sop*^+/?^) and *sop* homozygous embryos *(sop*^*−/−*^*)* at 5 dpf. *Sop*^*−/−*^ had an inducible wild type δ subunit gene, which was induced at 2 dpf resulting in partial paralysis [19]. Among the genes upregulated or downregulated in partially paralyzed embryos (Fig. 1A), a previously uncharacterized gene, *zgc153932* seemed to encode a membrane associated protein. Because we were interested in synaptic vesicle trafficking, we focused on this gene.

**Fig. 1:**
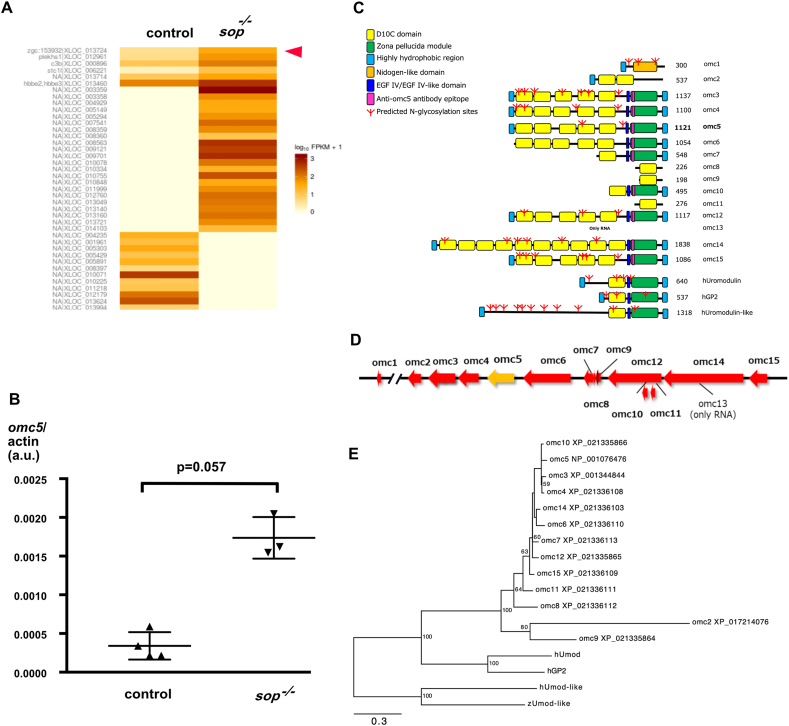
A. Heat map comparison of gene expression between control siblings (control) and partially paralyzed *sofa potato* mutants (*sop*^*−/−*^). *Omc5* gene is marked with an arrowhead. B. ddPCR of *omc5* transcript in control embryos (control) and paralyzed mutants (*sop*^*−/−*^) at 5 dpf. C. Diagram displaying protein products of *omcin 1-15* along with human *uromodulin*, human *GP2,* and human *uromodulin-like*. Domains/motifs are indicated with colored squares. D. A map of ∼400 KB region on Chromosome 12 where a cluster of *omcin* genes are located. E. Phylogenetic tree for the amino acid sequences of the zebrafish *omcin* genes, zebrafish *uromodulin-like* gene, human *uromodulin* gene, human *GP2* gene, and human *uromodulin-like* gene. Numbers in the nodes of the tree indicate bootstrap values.

Using droplet digital PCR analysis (ddPCR), the expression of *zgc153932* was examined in completely paralyzed *sop*^*−/−*^ embryos at 5 dpf. Consistent with the RNAseq data, the transcript was increased compared to wild type (Mann Whitney test; p = 0.057, Fig. 1B). At 2 dpf when the movement of wild type embryos are minimal, on the other hand, the expression of *zgc153932* was below the detection level, either for wild type or *sop*^*−/−*^ embryos. This suggests that the difference between *sop*^*−/−*^ embryos at 5 pdf is caused by phenotypes of *sop*^*−/−*^ embryos between 2 dpf and 5 dpf.

### 2.2 Genomic and structural characterization of *omc5*

To further characterize the *zgc153932* gene, we examined its product (Fig. 1C). The NetGPI program utilizing a deep learning approach (https://services.healthtech.dtu.dk/services/NetGPI-1.1/) predicted that it is GPI-anchored. It had five D10C domains, an EGF IV domain and a Zona Pellucida (ZP) module [[20], [21], [22]] near the C-terminus. Mammalian genes showing the highest sequence similarity to *zgc153932* were *uromodulin* and *GP2*. They both had a comparable overall structure with similar domains/modules: D10C, EGF IV and Zona pellucida.

Investigation of the zebrafish genome revealed that *zgc153932* had multiple paralogous genes in a region of ∼400 KB on the chromosome 12 (Fig. 1D). Based on their positions on the chromosome, we named these genes as *omcin 1* through *15*. These genes presumably comprise a gene family with high sequence similarities among themselves (supple.1). In this nomenclature, *zgc153932* is *omcin5 (omc5)*. We will henceforth use this designation in this manuscript.

The sequence similarity among these genes was generally high, particularly among those with amino acid lengths around 1100, namely *omcin 3, 4, 5, 6, 12* and *15*, while other genes had longer or shorter amino acid lengths (Fig. 1C). We generated a phylogenetic tree of the amino acid sequences for the *omcin* family genes along with related genes in zebrafish and human (Fig. 1E). Human genome has a *uromodulin-like* gene, which is a separate gene from *uromodulin*. Zebrafish also has a *uromodulin-like* gene, which is located on a separate chromosome, Chr 10. *Omcin1*, which was located 2 MB upstream from other *omcin* genes (Fig. 1D), was closer to human *uromodulin* or *GP2* genes than other *omcin* genes. Human genome does not have exactly matching genes of *omcin 2-15*, which are equally distant from human *uromodulin* and *GP2* (Fig. 1E).

### 2.3 Product of the *omc5* gene and its homologs

To further explore the function of *omc5* and its homologs, we generated an antibody against *omcin* family gene products, selecting a string of amino acids predicted to have high antigenicity as epitope (Fig. 2A). Since the epitope region was conserved among *omcin* family genes, this antibody was expected to recognize protein products of 6–9 *omcin* genes (Fig. 1C).

**Fig. 2:**
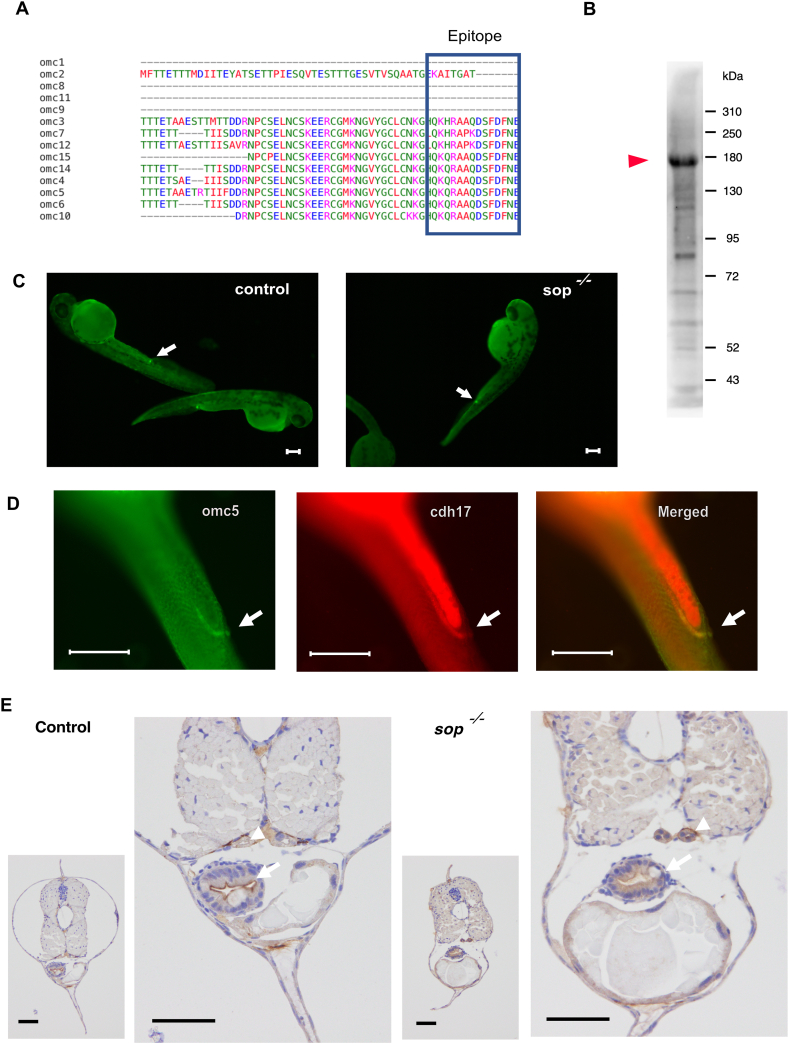
A. Amino acid sequence of *omcin* gene products surrounding the epitope used for the generation of antibody (boxed in blue). 6 of *omcin* genes including *omc5* have the identical sequence for the epitope, while additional 3 genes have close sequences. B. Western blot of the Omcin antibody on5dpf embryos. The strongest signal was observed around 180 kDa (arrowhead).C. Antibody signals observed near cloaca in control and *sop*^−/−^ embryos (arrows).D. Double staining in wild type embryos of Omcin antibody and *cdh17.* Signals indicated by arrows overlapped in the distal pronephros.E. Omcin antibody staining in slices of control and *sop*^−/−^embryos at 5 dpf. Signals in the gut (arrows) and the pronephros (arrowheads) are indicated. Scale bars, 50 μm.

In Western blot analysis of 5 dpf zebrafish embryos using the generated antibody, we detected a strongest band around 180 kDa, along with multiple bands at lower molecular weights (Fig. 2B). Six of the predicted molecular weights for *omcin* gene products (*omcin3, 4, 5, 6, 12* and *15*) are around 120 kDa without glycosylation, while others range from 55 kDa (*omc10*) to 207 kDa (*omc14*). Glycosylation occurs robustly in Uromodulin [[23], [24], [25]]. *Omcin* gene products also contained multiple glycosylation sites (Fig. 1C). Due to this complexity, we could not determine which of the observed bands correspond to *omc5*.

Using this antibody, we performed whole-mount immunohistochemistry (IHC) assays. At 1 dpf, the antibody signal was observed near the cloaca, where pronephros and intestine merge, in both control (*sop*^+/+^ or *sop*^+/−^) and *sop*^*−/−*^. The difference of signal intensity was not observed between the two groups (Fig. 2C). Using *cadherin-17 (cdh17)* as a marker of pronephros, we confirmed that the signal of the *omcin* antibody was in distal pronephros (Fig. 2D).

When we examined zebrafish larvae at 5 dpf, positive signals of the *omcin* antibody were observed in the epithelial cells of the renal tubule and the gut (Fig. 2E). The positive signal was not observed when the antibody was omitted in the reaction solution, confirming the specificity of the signal (supple. 2). The signal intensity in the kidney seemed stronger in *sop*^*−/−*^ embryos, while the signal in the gut seemed more diffuse and less restricted to the apical side.

### 2.4 Survival of embryos and *omc5* expression in the presence of *E. coli*

Based on these characteristics of *omcin* genes, we hypothesized that their functions are similar to that of *uromodulin* and *GP2*, whose encoded proteins bind to and control bacterial infections in urinary and intestinal tract [23,26].

**Fig. 3:**
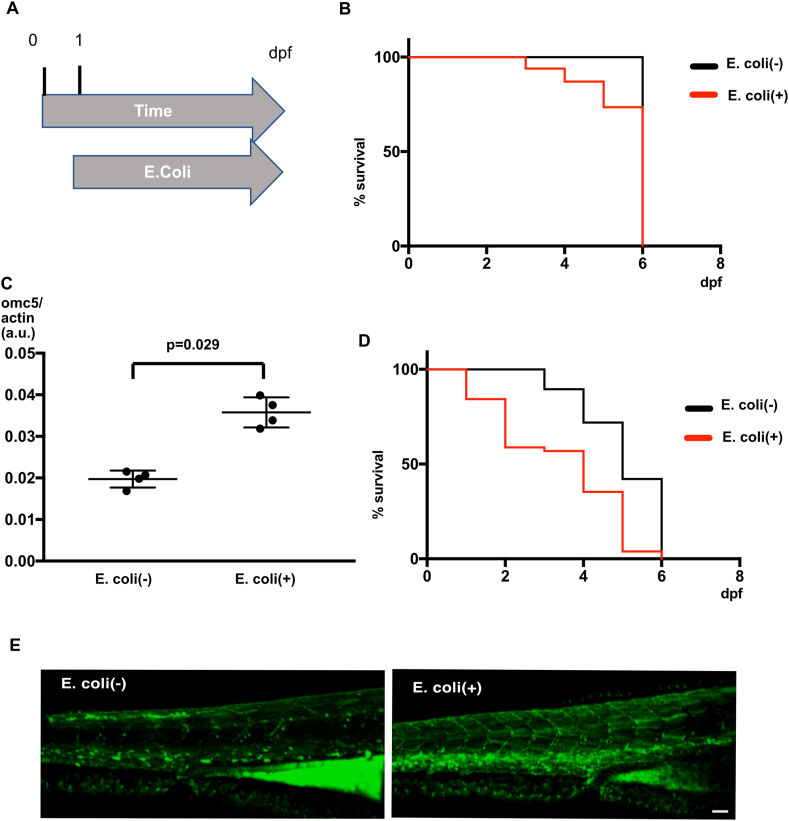
A. A time course of *E. coli* incubation experiment. Embryos were cultured in solution with or without *E. coli* from 1 dpf through 7 dpf. Surviving embryos were counted each day. B. Survival of wild type embryos were plotted against time in *E. coli* (−) or *E. coli* (+) solution. C. ddPCR of *omc5* in 5 dpf wild type embryos cultured in *E. coli* (−) or *E. coli* (+) solution. D. Survival of *sop*^−/−^embryos plotted against time in *E. coli* (−) or *E. coli* (+) solution. E. Neutrophils marked with GFP at 4 dpf embryos in *E. coli* (−) or *E. coli* (+) solution. 4 embryos were observed for each group and representative images are shown.

Zebrafish embryos were cultured in dishes with or without *E.coli* (NIHJ JC-2 strain) from 1 dpf through 7 dpf (see Methods. Fig. 3A). We counted the number of surviving embryos each day and calculated the percentage of surviving embryos. Wild type embryos survived 100 % in the *E.coli* (−) solution. In the *E.coli* (+) solution, the survival became shorter (Log-rank test, p < 0.001; Fig. 3B).

We examined the *omc5* expression level in response to the *E. coli* exposure. When ddPCR was performed on RNA extracted from 5 pdf wild type embryos, the expression of *omc5* was elevated in the group exposed to *E. coli* compared to controls (Mann Whitney test, p = 0.029; Fig. 3C).

We also examined the response of *sop*^*−/−*^ embryos to *E. coli*. Embryos started to die from 3 dpf in the *E.coli* (−) solution (Fig. 3D). In the *E.coli(+)* solution, the survival rate decreased further and got close to 0 % at 7 dpf (Log-rank test, p < 0.001).

In the *sop*^−/−^ embryos exposed to *E.coli*, neutrophils were visualized using 4 dpf mpx:GFP transgenic zebrafish embryos, which express GFP under the neutrophil-specific myeloperoxidase (*mpx*) promoter [27]. In embryos exposed to *E.coli*, neutrophils were more pronounced in the ventral trunk, compared to the control group (Fig. 3E). This indicates that *E. coli* exposure is able to induce an inflammatory response in zebrafish embryos.

### 2.5 *omc5* mutant zebrafish

**Fig. 4:**
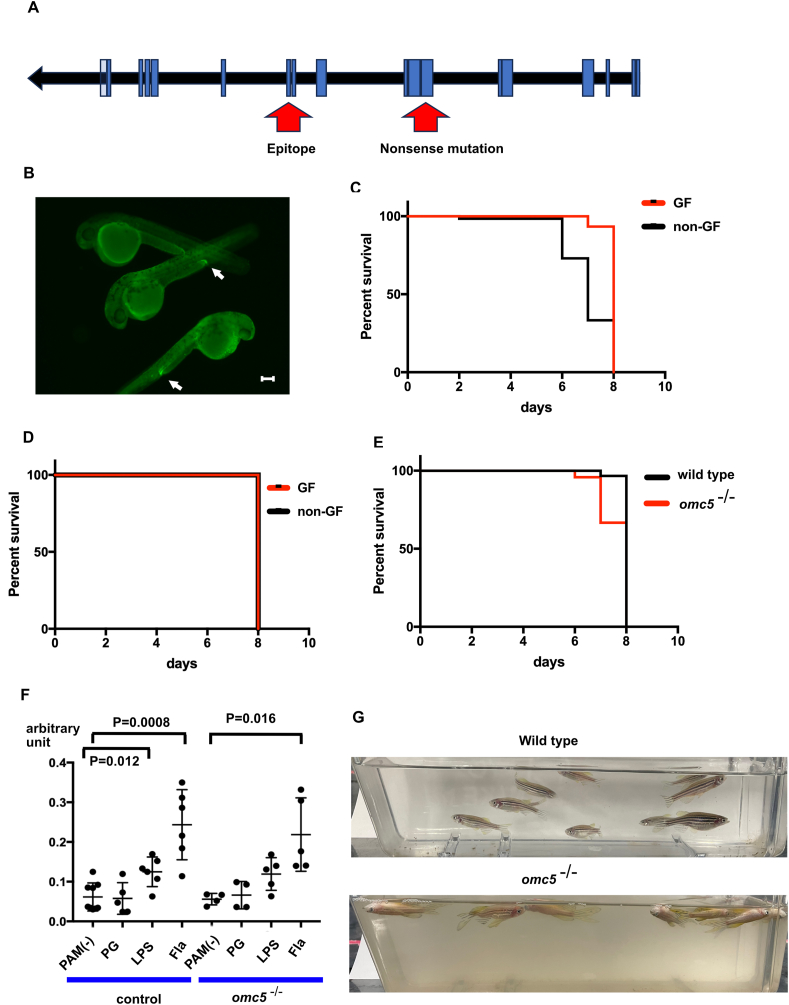
A. Diagram of *omc5* gene with exons indicated as blue boxes. Locations of nonsense mutation and the sequence encoding the epitope are indicated with arrows. B. Omcin antibody staining in *omc5*^−/−^ embryos. C. Survival of *omc5*^*−/−*^ embryos in germ-free solution (GF) and non germ-free solution (non-GF). D. Survival of wild type embryos in germ-free solution (GF) and non germ-free solution (non-GF). E. Survival of wild type and *omc5*^*−/−*^ embryos in *E.coli* (+) solution. F. *myd88* transcript measured by ddPCR in wild type (control) and *omc5*^−/−^ embryos after incubation in control solution (PAM(−)), Peptidoglycan (PG), Lipopolysaccharide (LPS) and Flagellin (Fla). G. 10 adult fish were housed in still water for 2 days. Wild type fish remained unaffected, while all *omc5*^*−/−*^ fish died.

To investigate the function of *omc5* further, we obtained an *omc5* KO zebrafish line from Zebrafish International Resource Center (ZIRC). The mutant allele harbored a premature stop codon in the 6th exon, which is upstream of the antibody recognition site (Fig. 4A). In the whole-mount IHC assay of *omc5*^−/−^ embryos, the antibody signal was detected near the cloaca in a similar fashion to wild type embryos, suggesting that the antibody recognized *omcin* gene products other than *omc5*, as expected (Fig. 1, Fig. 4B).

*Omc5*^−/−^ embryos started to die after 7 dpf when incubated in normal incubation condition. When embryos underwent “germ-free” procedure at 1 dpf (see Methods), the survival was extended (Log-rank, p < 0.01, Fig. 4C). The effect of germ-free procedure was not observed in wild type embryos (Fig. 4D). These results suggest that the shortening of life span in *omc5*^−/−^ embryos was caused by microorganisms contained in the fish maintenance system.

Microorganisms in the fish maintenance system likely fluctuate, and microbial composition is expected to be unstable. To examine susceptibility of *omc5*^−/−^ embryos to a single species of bacteria, *omc5*^−/−^ and wild type embryos after germ-free procedure were cultured in the MG1655 strain of *E.coli,* a strain that expresses type I fimbriae [28]. The survival was shortened in *omc5*^−/−^ embryos (Log-rank test, p < 0.01; Fig. 4E), suggesting that *omc5* confers resistance to microorganisms including *E.coli* with type I fimbriae.

We also explored a possibility that *omc5* is required in the signaling cascade initiated by pathogen associated molecular patterns (PAMPs). We exposed wild type (control) and *omc5*^−/−^ embryos to three PAMPs (peptidoglycan, lipopolysaccharide and flagellin) at 4 dpf and measured the *myd88* transcript with ddPCR (see Methods). MyD88 is positioned in multiple PAMP signaling cascades, and its transcript is often increased when the pathway is activated [29]. In our exposure protocols, wild type embryos showed increased transcripts of *myd88* in response to lipopolysaccharide and flagellin. *omc5*^−/−^ embryos also showed a similar pattern of *myd88* transcript change, which was not distinguishable from wild type embryos (Fig. 4F).

When *omc5*^−/−^ embryos grew to adults, they showed increased susceptibility to infection even housed in the circulating system equipped with multiple layers of mechanical filter and UV lamp. To analyze the susceptibility of adult fish to microorganisms, 5 males and 5 females aged between 6 months and 1 year were removed from the circulating maintenance system and placed in 500 mL of system water without circulation. After 2 days of incubation in still water, wild type fish did not show any sign of morbidity (Fig. 4G). In sharp contrast, all *omc5*^−/−^ fish died in two days (Fig. 4G). In summary *omc5*^−/−^ embryos and adults were compromised in their resistance to microorganisms.

### 2.6 *Omcin* genes in other teleosts

To examine the function of *omcin* genes from a different perspective, we examined a repertoire of *omcin* genes in other fishes. We screened the presence of *omcin* homologs in the teleost fishes by performing BLASTP search against the RefSeq Protein database. We used zebrafish Omc5 protein sequence as a query. Homologs of *omcins* were identified mainly in the Otocephala fish lineage. Putative *omcin* homologs were also found in some other fish species that belonged to Esociformes or Cyprinidontiformes. On the other hand, homologs of *omcins* were not detected in species of ancestral lineages, such as Anguilliformes, Osteoglossiformes, and Acipenseriformes.

We selected for phylogenetic analysis 6 teleost fishes (goldfish; *Carassius auratus*, electric eel; *Electrophorus electricus*, piranha; *Pygocentrus nattereri*, milkfish; *Chanos chanos*, channel catfish; *Ictalurus punctatus*, and European anchovy *Engraulis encrasicolus*), which were evolutionarily close to zebrafish and possessed genes homologous to zebrafish *omcin*s. Two fish species which were more distantly related to zebrafish (northern pike; *Esox lucius* and mummichog; *Fundulus heteroclitus*), were also included in the analysis because they had one or more putative *omcin* homologs. Homology searches (BLASTP) against the RefSeq protein sequence database in these fishes detected one or more copies of *omcin* homologs (Supple.3). Because homology search and multiple sequence alignment revealed that *omc1* did not show clear sequence homology to other *omcin*s, *omc1* was excluded from the dataset of phylogenetic analysis.

**Fig. 5:**
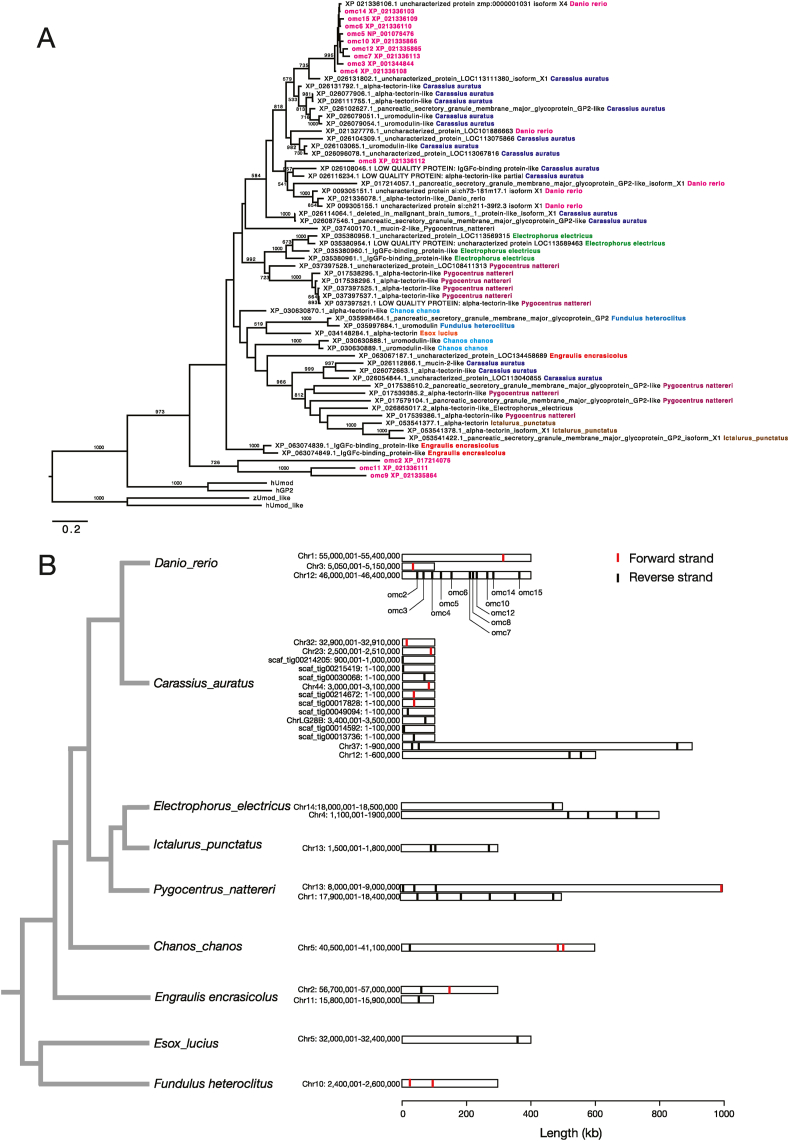
A. A maximum-likelihood phylogenetic tree of the zebrafish *omcin*s and *omcin*-like genes found in the 6 fishes (goldfish, electric eel, piranha, milkfish, northern pike, channel catfish, European anchovy, and mummichog). Numbers in each tree node indicate bootstrap values (only the values > 50 % are shown). B. Chromosomal locations of the *omcins* in zebrafish and *omcin*-like genes in the 8 fishes examined. Phylogenetic relationship of these fishes is also shown. Vertical lines indicate the start codon positions. Transcriptional orientations are shown by colors (red: forward strand, black: reverse strand).

A maximum-likelihood tree of the zebrafish *omcin*s and *omcin* homologs in the 8 fishes showed that 9 zebrafish *omcin*s (*omc14, 3, 15, 5, 6, 10, 4, 12*, and *7*) formed a monophyletic clade with a high (99.5 %) bootstrap support (Fig. 5A). One goldfish *omcin* homolog (XP_026131802) was clustered with the zebrafish *omcin* clade (Fig. 5A), suggesting the orthologous relationship between the XP_026131802 gene and 9 zebrafish *omcins*. Three zebrafish *omcin*s (*omc2, 9*, and *11*) formed another clade (Fig. 5A), but orthologs of these *omcin*s were not found from the 8 fishes examined in this study. *Omc8*, was not clustered with the other *omcin*s and *omcin* homologs in the 8 fishes (Fig. 5A).

Our phylogenetic analysis also showed that the *omcin* homologs in the 8 fishes tend to form monophyletic clades within each species (Fig. 5A). This implies that duplications and losses of *omcin* genes occurred frequently in the teleost fish lineage. For example, clades that consist of 4 or more *omcin* homologs were identified not only in zebrafish, but also in goldfish, piranha, and electric eel. *Omcin* genes in these species were located within a small genomic region as gene clusters (Fig. 5B). Notably, aligned sequence regions (mainly ZP module) of the 9 zebrafish *omcin* paralogs were very similar (Fig. 1, Supple.1), suggesting that the zebrafish *omcin*s have expanded their copies in a short evolutionary period of time.

## 3 Discussion

In this study we characterized the *omcin* gene family in zebrafish, one of which showed increased expression with bacterial exposure. Because structurally related human proteins Uromodulin and GP2 bind to bacteria and control infection, we hypothesized that *omcin* genes have a similar function.

We reason that the increase of *omc5* in paralyzed *sop*^−/−^embryos resulted from bacteria in the incubation solution. Bacterial infection in zebrafish is achieved in experiments by multiple methods including immersion, injection into the duct of Cuvier [7], or microgavage [10]. In *sop*^−/−^, bacteria in the surrounding solution presumably infiltrated the embryos due to the immobility of embryos.

In the RNAseq analysis (Fig. 1A; data availability statement), there were two prominent groups of genes upregulated in *sop*^−/−^. One included genes implicated in hypoxia, and the other comprised those related to infection/inflammation. In the former group were *ankyrin repeat domain 37*, *myoglobin*, *hemoglobin beta embryonic-2*, *hemopexin b*, *egl-9 family hypoxia-inducible factor 3*, *pyruvate dehydrogenase kinase isoform2* and *erythrocyte membrane band 4.1b*. It is reasonable to expect hypoxia in paralyzed mutants, which supports the validity of the RNAseq data. The latter included *complement component 3*, *immunoresponsive gene1-like*, *myeloid specific peroxidase* and *intelectin 3*. Upregulation of these genes complies with our hypothesis that *omc5* is involved in the response of zebrafish to bacterial infection.

We cultured embryos containing *E. coli* marked with mCherry from 1 dpf through 4 dpf, and observed their fluorescence. While mCherry signals were occasionally observed on epithelial cells lining the gut, clear colonization of *E. coli* was not observed in wild type or *sop*^−/−^ embryos. This may be due to the absence of *E. coli* in the natural microbiota of zebrafish gut [30]. Although we lack direct evidence, we speculate that reduced water flow around cloaca in paralyzed embryos lead to easier infiltration of bacteria. Localization of *omcin* proteins in the renal tubule and the gut as well as the increased neutrophils in *E. coli* (Fig. 3E) are in line with this hypothesis.

In zebrafish, *omcin* genes constitute a gene cluster, forming clades in the phylogenetic tree clearly distinguished from *uromodulin* or *GP2* (Fig. 1E). Notably, the number of D10C modules was highly variable in *omcin* gene products (Fig. 1C). While regions close to the C-termini in Omc5 had higher sequence similarities, the overall identity of Omc5 to Uromodulin and GP2 was 30.3 % and 31.4 % respectively. Zebrafish genome therefore does not have *uromodulin* nor *GP2*, and *omcin* genes constitute family genes distinct from *uromodulin* or *GP2*.

Zebrafish *uromodulin-like* is distinct from *omcin* family genes and forms a clade with human *uromodulin-like* (Fig. 1E). The gene described as *GP2-like* in NCBI (LOC100005685) is *omcin7* in our nomenclature.

The lack of unrelated genes in the ∼400 KB region containing *omcin* family genes is notable. Similar genomic compositions are reported in family genes such as Hox [31], rhodopsin [32], and apolipoprotein genes [33]. Comparison of amino acid length and domains/modules suggest that *omcin3*, *4, 5, 6, 12* and *15* are *bona fide* cluster genes (Fig. 1C, D, E). *Omcin1* is structurally distant and is located ∼2 MB upstream of other *omcin* genes, rendering it an unlikely member of the cluster. *Omcin 2, 8, 9, 10,* and *11* are much shorter, with amino acid sequence 200–600, while *omcin14* has around ∼1800 amino acids (Fig. 1C). These lengths as well as the phylogenetic analysis make *omcin 2, 8, 9* and *11* possible outliers among the family (Fig. 1, Fig. 5A).

In a recent study, Weiss et al. showed that human Uromodulin binds to multiple bacterial species [23]. They identified individual N-glycans attached to asparagine (Asn) residues of human Uromodulin, among which only Asn275 had capability to bind to *E.coli.* Human Uromodulin aggregated not only *E. coli* but also other bacteria found in urinary tract such as *Klebsiella pneumoniae, Pseudomonas aeruginosa, and Streptococcus mitis.* Omc5, like Uromodulin, may bind to multiple bacteria. The upregulated expression of *omc5* transcripts in paralyzed *sop*^*−/−*^ embryos (Fig. 1) as well as in *E. coli* (+) wild type embryos (Fig. 3C) and the susceptibility of *omc5*^*−/−*^ mutants to microorganisms (Fig. 4) suggested its involvement in infection.

Further search in literature revealed that *omc5* is also increased in response to *Shigella* injected into the yolk sack of zebrafish [7]. Moreover, *omc5* transcription was strongly upregulated when germ-free zebrafish are colonized with *Chryseobacterium* sp. (4.81 fold) or *Exiguobacterium* sp. (9.66 fold) [2]. The increased transcript of *omc5* in response to such a broad spectrum of bacterial suggests the role of this gene and perhaps the larger gene family in immune response.

The survival of *omc5*^−/−^ embryos was shortened in non-germ-free solution or in *E. coli* (Fig. 4C–E), while the response of *myd88* to PAMPs was not affected (Fig. 4F). Therefore, *omc5* gene function is required for the response of host animal to microorganisms, while the response to PAMPs tested in this study was independent from *omc5*. Because type I fimbriae bind to Uromodulin [24], it is likely that Omc5 protein binds to bacteria with type I fimbriae.

The strong phenotypes in *omc5*^*−/−*^ mutants (Fig. 4) were unexpected, because *omcin* family genes with similar protein structures are present in *omc5*^−/−^ mutants. Individual *omcin* genes may have overlapping yet distinct functions. Further characterization of *omcin* genes in future studies will provide more information. Knocking out multiple *omcin* genes may also be possible due to the proximity of *omcin* genes on the chromosome (Fig. 1D).

Our phylogenetic analysis showed that the orthologous relationship of the 9 zebrafish *omcins* and one goldfish *omcin* homologs, XP_026131802 (Fig. 5A). This indicates that the expansion of the zebrafish *omcin*s occurred after the separation of zebrafish and goldfish lineages. In the teleost fishes possessing *omcin*-like genes, the copy numbers of *omcin* genes were highly variable, ranging from 1 (northern pike) to over 10 (zebrafish and goldfish) (Fig. 5). This implies that, in teleost fishes, *omcin* genes evolved with frequent gene duplications/losses, as is observed in evolution of complement 3, regulatory factor H, or cathelicidin genes [34,35] Diversification of these immune-related genes in teleost fishes occurred in a microbial-rich aquatic environment compared to terrestrial vertebrates [34]. The diverse *omcin* gene repertoires in fishes may result from adaptation to natural habitats for each species. Multiple environmental factors such as temperature, salinity, and nutrients in the water lead to different combinations of pathogenic bacteria. Thus, the repertoire of *omcin* genes in an individual species may be optimized to resist a specific combination of environmental pathogens. Comparison of bacterial composition in the natural habitat and repertoire of *omcin* genes in multiple fish species will be an attractive topic of future studies.

## 4 Materials and methods

### 4.1 Fish strains

*Sofa potato* mutant (tj^19d^) was originally generated in the ENU mutagenesis screening [15]. Adult fish were maintained in a stand-alone, self-circulating Tecniplast system following Institutional Animal Care and Use Committee guidelines at OMPU (#AM23-026). *mpx:GFP* transgenic fish (uwm1Tg)and *omc5* mutant fish (sa22172)were obtained from ZIRC, University of Oregon. All embryos were reared at 28 °C in egg water under 14hr-light 10hr-dark light cycle.

### 4.2 RNA-seq

Zebrafish embryos obtained from a cross of *sop* ^±^ were separated into the paralyzed homozygous group (*sop*^−/−^) and the swimming siblings (*sop*^+/+^ or *sop*^+/−^). Both groups were subjected to mifepristone to induce wild type δsubunit to partially rescue paralyzed fish [19]. Total RNA was extracted from both groups (∼10 larvae in each) at 5 dpf using RNeasy Mini Kit (QIAGEN). Next-Generation sequencing and bioinformatics analysis was performed using TopHat and Cufflinks by Zymo Research. Read counts were ∼56M and ∼144M for *sop*^−/−^ and controls respectively.

### 4.3 DNA extraction and droplet digital PCR

Total RNA was isolated from 2 dpf or 5 dpf zebrafish embryos using Nucleospin RNA XS (Macherey-Nagel) and reverse transcribed with PrimeScript RT reagent kit (Takara) according to the manufacturer's instructions. The assay was performed using the Bio-Rad QX200 Droplet Digital System (Bio-Rad Laboratories). 1 μL cDNA samples, 1 μL primer/probe mixture for *omc5, myd88* or *actb1* (designed and synthesized by Bio-Rad; Supple.4), 10 μL 2× ddPCR supermix for probes, and 8 μLRNase-free water were mixed for each 20 μL reaction mixture. Droplets were then generated by a QX200 droplet generator device (Bio-Rad). The cycling conditions were as follows: 10min at 95 °C, 40 cycles of denaturation at 94 °C for 15 s, annealing and extension for 1 min, and final step at 98 °C for 10 min. The annealing temperature was 52 °C for *omc5* and 54 °C for *myd88* and *actb1*. Droplets were read in the droplet reader and analyzed using QuantaSoft (Bio-Rad Laboratories). Transcripts of *omc5* or *myd88* were normalized by those of *actb1*.

### 4.4 Bioinformatics

Refseq protein sequences of 8 fishes used for the analysis were downloaded from the NCBI database (https://www.ncbi.nlm.nih.gov/). To identify *omcin* homologs in these fishes, BLASTP searches were conducted against their protein sequence datasets using zebrafish Omc10 as a query. Chromosomal locations of the *omcin* homologs were confirmed by the genome sequences of these fishes using NCBI Genome Data Viewer (https://www.ncbi.nlm.nih.gov/gdv/?org=engraulis-encrasicolus). Amino acid sequences of the zebrafish *omcin*s and detected *omcin* homologs in the 8 fishes were aligned using MAFFT v7.4.9.0 [36]. Phylogenetic trees of *omcin*s were constructed by the maximum-likelihood method with Jones-Taylor-Thornton (JTT) model using MEGA 11 (version 11.0.13) software package [37]. To include all zebrafish *omcin* sequences in the phylogenetic tree, a "partial deletion" option implemented in MEGA11 was used. A cutoff of the proportion of aligned sequences was set to 50 %. Human and zebrafish *uromodulin-like* (Umod-like), a human *uromodulin* (hUmod), and a human *GP2* (hGP2) were used as outgroup sequences. Reliability of the tree nodes were assessed by the bootstrap method with 1000 replications.

To identify domains and motifs, a prediction of the three-dimensional structure of *omcin 5* (zgc153932) was obtained from the AlphaFold Protein Structure Database (https://www.alphafold.ebi.ac.uk/entry/A2RUV7). Other *omcin* protein structures were predicted by ColabFold v1.5.5 (https://github.com/sokrypton/ColabFold). PyMOL version 1.4.1 (https://www.pymol.org/) was used to visualize structures.

### 4.5 Antibody generation and Western blotting

Antibody was generated in rabbits against the epitope KQRAAQDSFDFNE (Eurofin). 5dpf embryos were lysed using ice-cold RIPA buffer (Tris 10 mM pH 7.6, NaCl 150 mM, Sodium Dodecyl Sulfate (SDS) 0.1 %, Nonidet P-40 1 %, and Protease and Phosphatase Inhibitor Cocktail (Thermo Fisher Scientific)), followed by a brief grinding using disposal homogenizer (BioMasher®) and centrifugation for 15 min at 1000 g and 4 °C to remove debris. Protein concentrations were determined using the bicinchoninic acid (BCA) protein assay kit (Thermo Fischer Scientific). For Western blot analysis, samples were denatured by heating for 5 min at 95 °C using DTT. Samples were diluted in 6× sample buffer (Nacalai), separated on a 7.5 % SDS-PAGE gel and blotted onto methanol-activated PVDF membranes. Membranes were blocked for 1 h in 5 % w/v non-fat dry milk solution at room temperature, followed by overnight incubation at 4 °C with primary antibody (Eurofin) at 1:1500 dilution. Thereafter, blots were washed and incubated with HRP-conjugated Goat anti Rabbit antibody (Proteintech) at 1:5000 dilution, washed again and visualized by Western BLoT Quant HRP Substrate (Takara).

### 4.6 Immunohistochemistry

1 dpf anesthetized zebrafish embryos were fixed for 24 h in 4 % paraformaldehyde in T-PBS. After fixation and rinsing with T-PBS for 10 min, the embryos were immersed and permeabilized in cold 100 % acetone for 7 min at −20 °C. Thereafter, samples were washed for 10 min with T-PBS. Samples were subsequently blocked and stained with the Omcin antibody at 1:500 dilution in blocking solution (2 % goat serum (Abcam) in T-PBS) and kept overnight at 4 °C. After washing for 15 min in T-PBS, secondary antibody (Goat Anti-rabbit Alexa 488 antibody, Thermofisher) was added at 1:1000 dilution, and kept for 3 h at room temperate. Samples were washed for 1 h in T-PBS and observed by fluorescence microscope (BZ-x700, KEYENCE).

For co-staining assay, RNA probe was generated using the sequence of *cdh17* gene as the template. *cdh17* cDNA was generated by PCR using gene specific primers with SP6/T7 promoters attached (Supple.4). Using the PCR product, DIG RNA Labeling [SP6/T7] (Roche) was performed according to the manufacturer's instructions.

1 dpf anesthetized zebrafish embryos were rinsed with 75 %, 50 %, 25 % methanol/T-PBS each for 5 min, followed by immersion in Proteinase K in T-PBS for 10 min. The embryos were fixed for 20 min in 4 % paraformaldehyde in T-PBS. After fixation, the embryos were rinsed with T-PBS for 25 min, and immersed in hybridization solution for 2 h at 65°. After denaturing in hybridization solution for 10 min at 80 °C, the denatured RNA probe was added to the samples and hybridized for 24 h at 65 °C. Samples were washed for 1 h with 2× SSCT (0.3 M NaCl, 30 mM sodium citrate, 0.1 % Tween-20)/50 % formamide, 2× SSCT, 0.2× SSCT. After blocking with 5 % normal sheep serum (Sigma) in T-PBS for 2 h, samples were treated with the Omcin antibody at 1:500 dilution in the blocking solution, and kept overnight at 4 °C. After washing for 15 min in T-PBS, samples were treated with Goat Anti-rabbit Alexa 488 antibody (Thermo fisher) at 1:1000 dilution and anti-DIG-Alkaline Phosphatase antibody (Sigma) at 1:2500 dilution, overnight at 4 °C. Samples were washed for 2 h in T-PBS and stained with staining buffer (100 mM Tris-HCl (pH 9.5), 100 mM NaCl) for 10 min, by HNPP/Fast Red TR (HNPP Fluorescent Detection Set, Roche) for 1 h, and observed by fluorescence microscope (BZ-X700, KEYENCE).

For slice preparation at 5 dpf, zebrafish embryos were fixed in 10 % neutral buffered formalin for 24 h at room temperature, and embedded in paraffin wax. The formaldehyde-fixed and paraffin-embedded zebrafish sections on glass slides were deparaffinized and hydrated, subsequently stained with the Omcin antibody at 1:4000 dilution with BOND Polymer Refine Detection (cat. no. DS9800; Leica Microsystems) and BOND-MAX (Leica Microsystems) according to the manufacturer's instructions, in which staining was performed using 3,3′-Diaminobenzidine tetrahydrochloride hydrate to visualize the antibody signal via brown precipitate.

### 4.7 Incubation of embryos in *E. Coli* and PAMPs

*E.coli* (NIHJ JC-2 or MG1655 strain) in LB medium was cultured overnight in a 37 °C shaker. OD600 was measured with SmartSpec3000 (Bio-rad). If the obtained value was <0.20 in 10X dilution, the experiment was aborted. LB solution containing *E.coli* was added to 20 mL of egg water so that the final concentration was 0.03 (Fig. 3) or 0.015 (Fig. 4) OD600. For control, LB solution without *E.coli* was added. Embryos displaying normal development were selected on 1 dpf and used for observing survival up to 7 or 9 dpf. Incubation solution was not changed throughout the incubation period. Embryos were judged dead when the heart beats were not observed.

To visualize neutrophils in zebrafish with GFP, *mpx:GFP* embryos after PTU treatment were observed under confocal microscope embryos at 4 dpf. PTU treatment was performed as previously described [38].

To culture embryos in germ-free condition, we followed a procedure of germ-free derivation [39]. Fertilized eggs were collected and bathed in egg water containing Ampicillin, Kanamycin and Amphotericin B (100 μg/mL, 5 μg/mL and 250 ng/mL final concentration respectively) for 4 h. After treatment in egg water containing 0.003 % NaHCl for 20 min, eggs were transferred to egg water filtered through a 0.2 μm filter.

For exposure to PAMPs, peptidoglycan from *Staphylococcus aureus* (#77140-10 MG, Sigma-Aldrich), lipopolysaccharide from *Salmonella minnesota* (#304, Funakoshi) and Flagellin from *Salmonella typhimurium* (#AG-40B-0025-C010, Funakoshi) were dissolved at 1 μg/mL, 100 μg/mL and 1.7 μg/mL final concentration respectively, and embryos were bathed in the solution at 4 dpf. After exposure for 24 h, embryos were harvested for RNA extraction. After reverse transcription, ddPCR was performed using primers listed in Supple.4.

### 4.8 Statistics

Statistical analysis with one-way ANOVA and/or Mann-Whitney test was performed with PRISM. Survival was analyzed with Log-rank test.

## Data availability statement

Data for Fig. 1A can be downloaded at https://datadryad.org/stash/share/5SeeNhzO8cn0tM2QOMnEWkmfC9MkvGbOKV7vH38pxvU.

## Ethics statement

All animal experiments were approved by the Institutional Animal Care and Use Committee guidelines at OMPU (#AM23-026).

## CRediT authorship contribution statement

**Shiori Naruoka:** Writing – original draft, Investigation, Conceptualization. **Souhei Sakata:** Supervision, Investigation, Conceptualization. **Shigeru Kawabata:** Investigation. **Yasuyuki Hashiguchi:** Investigation. **Eriko Daikoku:** Investigation. **Shoichi Sakaguchi:** Investigation. **Fumiyoshi Okazaki:** Resources. **Kento Yoshikawa:** Investigation. **John F. Rawls:** Writing – review & editing. **Takashi Nakano:** Resources. **Yoshinobu Hirose:** Investigation. **Fumihito Ono:** Writing – review & editing, Supervision, Investigation, Conceptualization.

## Declaration of competing interest

The authors declare the following financial interests/personal relationships which may be considered as potential competing interests:Fumihito Ono reports financial support was provided by 10.13039/501100001691Japan Society for the Promotion of Science. If there are other authors, they declare that they have no known competing financial interests or personal relationships that could have appeared to influence the work reported in this paper.

## Figures and Tables

**figs1:**
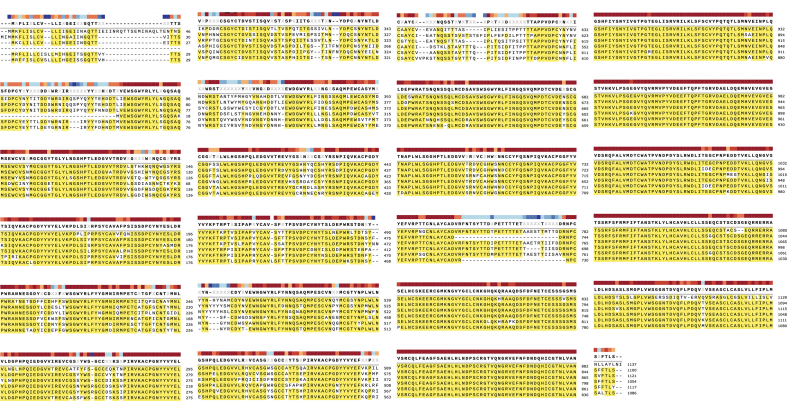


**figs2:**
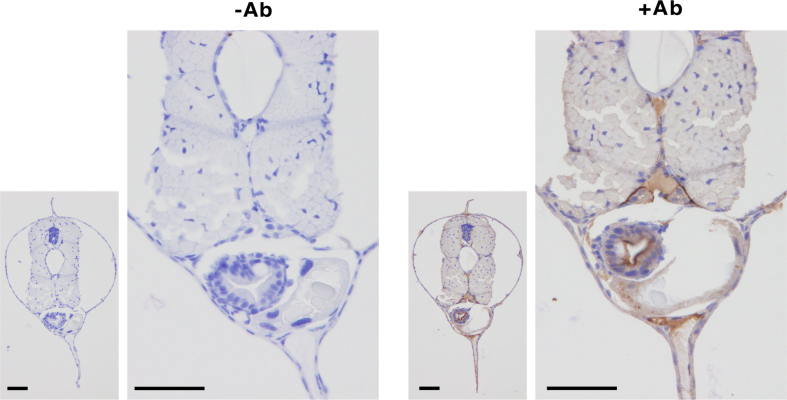


## References

[1] Kanther M., Rawls J.F. (2010). Host–microbe interactions in the developing zebrafish. Curr. Opin. Immunol..

[2] Koch B.E.V., Yang S., Lamers G., Stougaard J., Spaink H.P. (2018). Intestinal microbiome adjusts the innate immune setpoint during colonization through negative regulation of MyD88. Nat. Commun..

[3] Mostowy S., Boucontet L., Moya M.J.M., Sirianni A., Boudinot P., Hollinshead M. (2013). The zebrafish as a new model for the in vivo study of Shigella flexneri interaction with phagocytes and bacterial autophagy. PLoS Pathog..

[4] Varas M., Fariña A., Díaz-Pascual F., Ortíz-Severín J., Marcoleta A.E., Allende M.L. (2017). Live-cell imaging of Salmonella Typhimurium interaction with zebrafish larvae after injection and immersion delivery methods. J. Microbiol. Methods.

[5] Willms R.J., Jones L.O., Hocking J.C., Foley E. (2022). A cell atlas of microbe-responsive processes in the zebrafish intestine. Cell Rep..

[6] Di Q., Lin Q., Huang Z., Chi Y., Chen X., Zhang W. (2017). Zebrafish nephrosin helps host defence against Escherichia coli infection. Open Biol..

[7] Torraca V., Kaforou M., Watson J., Duggan G.M., Guerrero-Gutierrez H., Krokowski S. (2019). Shigella sonnei infection of zebrafish reveals that O-antigen mediates neutrophil tolerance and dysentery incidence. PLoS Pathog..

[8] Stockhammer O.W., Rauwerda H., Wittink F.R., Breit T.M., Meijer A.H., Spaink H.P. (2010). Transcriptome analysis of Traf6 function in the innate immune response of zebrafish embryos. Mol. Immunol..

[9] Li X., Wang S., Qi J., Echtenkamp S.F., Chatterjee R., Wang M. (2007). Zebrafish peptidoglycan recognition proteins are bactericidal amidases essential for defense against bacterial infections. Immunity.

[10] Ye L., Bae M., Cassilly C.D., Jabba S.V., Thorpe D.W., Martin A.M. (2021). Enteroendocrine cells sense bacterial tryptophan catabolites to activate enteric and vagal neuronal pathways. Cell Host Microbe.

[11] Bernut A., Moigne V.L., Lesne T., Lutfalla G., Herrmann J.-L., Kremer L. (2014). In vivo assessment of drug efficacy against Mycobacterium abscessus using the embryonic zebrafish test system. Antimicrob. Agents Chemother..

[12] Saraceni P.R., Romero A., Figueras A., Novoa B. (2016). Establishment of infection models in zebrafish larvae (Danio rerio) to study the pathogenesis of Aeromonas hydrophila. Front. Microbiol..

[13] Rauta P.R., Nayak B., Das S. (2012). Immune system and immune responses in fish and their role in comparative immunity study: a model for higher organisms. Immunol. Lett..

[14] Matthews J.L. (2004). Common diseases of laboratory zebrafish. Methods Cell Biol..

[15] Ono F., Higashijima S., Shcherbatko A., Fetcho J.R., Brehm P. (2001). Paralytic zebrafish lacking acetylcholine receptors fail to localize rapsyn clusters to the synapse. J. Neurosci..

[16] Devuyst O., Olinger E., Rampoldi L. (2017). Uromodulin: from physiology to rare and complex kidney disorders. Nat. Rev. Nephrol..

[17] Hase K., Kawano K., Nochi T., Pontes G.S., Fukuda S., Ebisawa M. (2009). Uptake through glycoprotein 2 of FimH+ bacteria by M cells initiates mucosal immune response. Nature.

[18] Ono F., Mandel G., Brehm P. (2004). Acetylcholine receptors direct rapsyn clusters to the neuromuscular synapse in zebrafish. J. Neurosci..

[19] Mott M., Luna V.M., Park J.-Y., Downes G.B., Epley K., Ono F. (2017). Expressing acetylcholine receptors after innervation suppresses spontaneous vesicle release and causes muscle fatigue. Sci. Rep..

[20] Bokhove M., Nishimura K., Brunati M., Han L., Sanctis D de, Rampoldi L. (2016). A structured interdomain linker directs self-polymerization of human uromodulin. Proc. Natl. Acad. Sci. USA.

[21] Stsiapanava A., Xu C., Brunati M., Zamora‐Caballero S., Schaeffer C., Bokhove M. (2020). Cryo‐EM structure of native human uromodulin, a zona pellucida module polymer. EMBO J..

[22] Han L., Monné M., Okumura H., Schwend T., Cherry A.L., Flot D. (2010). Insights into egg coat assembly and egg-sperm interaction from the X-ray structure of full-length ZP3. Cell.

[23] Weiss G.L., Stanisich J.J., Sauer M.M., Lin C.-W., Eras J., Zyla D.S. (2020). Architecture and function of human uromodulin filaments in urinary tract infections. Science.

[24] Pak J., Pu Y., Zhang Z.-T., Hasty D.L., Wu X.-R. (2001). Tamm-horsfall protein binds to type 1 fimbriated Escherichia coli and prevents E. coli from binding to uroplakin ia and ib receptors. J. Biol. Chem..

[25] van Rooijen J.J.M., Voskamp A.F., Kamerling J.P., Vliegenthart J.F.G. (1999). Glycosylation sites and site-specific glycosylation in human Tamm-Horsfall glycoprotein. Glycobiology.

[26] Stsiapanava A., Xu C., Nishio S., Han L., Yamakawa N., Carroni M. (2022). Structure of the decoy module of human glycoprotein 2 and uromodulin and its interaction with bacterial adhesin FimH. Nat. Struct. Mol. Biol..

[27] Hall C., Flores M.V., Chien A., Davidson A., Crosier K., Crosier P. (2009). Transgenic zebrafish reporter lines reveal conserved Toll‐like receptor signaling potential in embryonic myeloid leukocytes and adult immune cell lineages. J. Leukoc. Biol..

[28] Gally D.L., Bogan J.A., Eisenstein B.I., Blomfield I.C. (1993). Environmental regulation of the fim switch controlling type 1 fimbrial phase variation in Escherichia coli K-12: effects of temperature and media. J. Bacteriol..

[29] Tang D., Kang R., Coyne C.B., Zeh H.J., Lotze M.T. (2012). PAMPs and DAMPs: signal 0s that spur autophagy and immunity. Immunol. Rev..

[30] Wong S., Stephens W.Z., Burns A.R., Stagaman K., David L.A., Bohannan B.J.M. (2015). Ontogenetic differences in dietary fat influence microbiota assembly in the zebrafish gut. mBio.

[31] Amores A., Force A., Yan Y.-L., Joly L., Amemiya C., Fritz A. (1998). Zebrafish hox clusters and vertebrate genome evolution. Science.

[32] Kakarala K.K., Jamil K. (2014). Sequence-structure based phylogeny of GPCR Class A Rhodopsin receptors. Mol. Phylogenet. Evol..

[33] Gu L., Xia C. (2019). Cluster expansion of apolipoprotein D (ApoD) genes in teleost fishes. BMC Evol. Biol..

[34] Najafpour B., Cardoso J.C.R., Canário A.V.M., Power D.M. (2020). Specific evolution and gene family expansion of complement 3 and regulatory factor H in fish. Front. Immunol..

[35] Maier V.H., Dorn K.V., Gudmundsdottir B.K., Gudmundsson G.H. (2008). Characterisation of cathelicidin gene family members in divergent fish species. Mol. Immunol..

[36] Katoh K., Standley D.M. (2013). MAFFT multiple sequence alignment software version 7: improvements in performance and usability. Mol. Biol. Evol..

[37] Tamura K., Stecher G., Kumar S. (2021). MEGA11: molecular evolutionary genetics analysis version 11. Mol. Biol. Evol..

[38] Fujii K., Nakajo K., Egashira Y., Yamamoto Y., Kitada K., Taniguchi K. (2020). Gastrointestinal neurons expressing HCN4 regulate retrograde peristalsis. Cell Rep..

[39] Pham L.N., Kanther M., Semova I., Rawls J.F. (2008). Methods for generating and colonizing gnotobiotic zebrafish. Nat. Protoc..

