# Supplementary material for: A zebrafish gene with sequence similarities to human uromodulin and GP2 displays extensive evolutionary diversification among teleost and confers resistance to bacterial infection
Source: Heliyon. 2024 Sep 6;10(18):e37510. doi: 10.1016/j.heliyon.2024.e37510 (PMC11415648; doi:10.1016/j.heliyon.2024.e37510)
Supplement: Multimedia component 2 [file mmc2.pptx]

## Slide 1
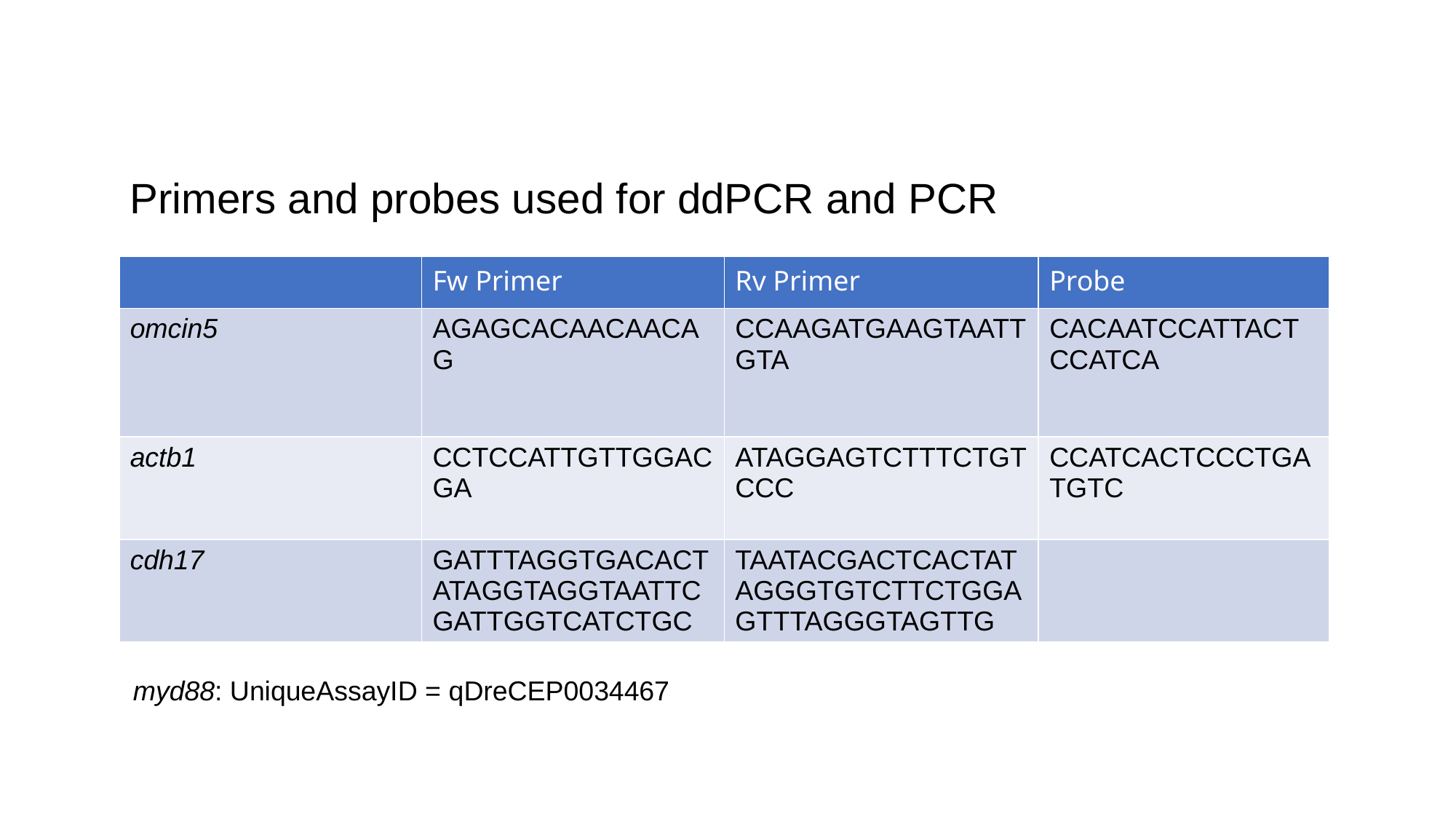

# Primers and probes used for ddPCR and PCR
| | Fw Primer | Rv Primer | Probe |
| --- | --- | --- | --- |
| omcin5 | AGAGCACAACAACAG | CCAAGATGAAGTAATTGTA | CACAATCCATTACTCCATCA |
| actb1 | CCTCCATTGTTGGACGA | ATAGGAGTCTTTCTGTCCC | CCATCACTCCCTGATGTC |
| cdh17 | GATTTAGGTGACACTATAGGTAGGTAATTCGATTGGTCATCTGC | TAATACGACTCACTATAGGGTGTCTTCTGGAGTTTAGGGTAGTTG | |
myd88: UniqueAssayID = qDreCEP0034467
